# Supplementary material for: The interactome of intact mitochondria by cross-linking mass spectrometry provides evidence for coexisting respiratory supercomplexes
Source: Mol Cell Proteomics. 2017 Dec 8;17(2):216–32. doi: 10.1074/mcp.RA117.000470 (PMC5795388; doi:10.1074/mcp.RA117.000470)
Supplement: Supplemental Data [file supp_RA117.000470_133922_0_supp_23978_fzffwf.zip › spectra_annotation/mito_spectra_annotation/392-1-22-1-8-1.pdf]

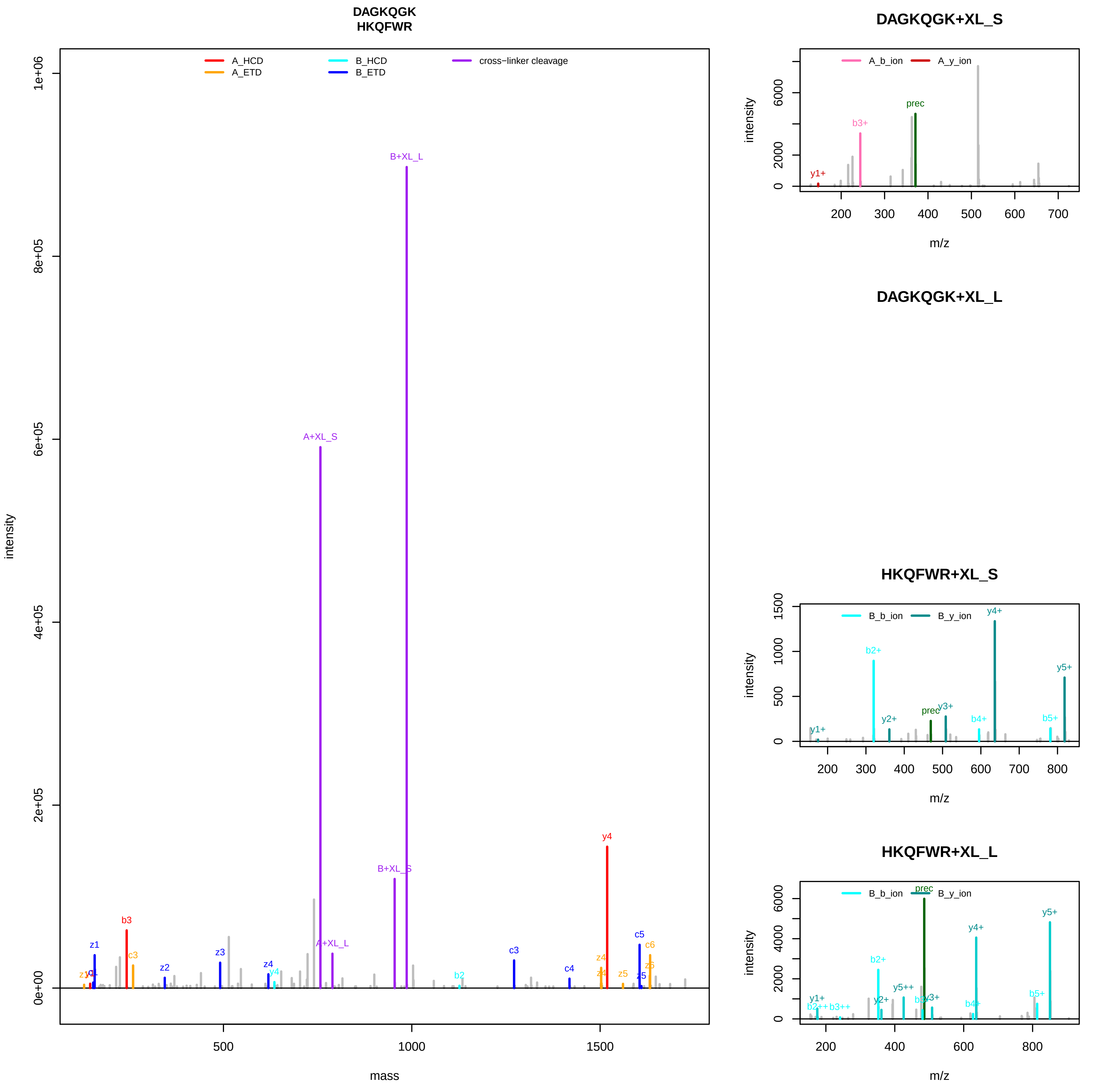

DAGKQGK
HKQFWR
A_HCD
A_ETD
B_HCD
B_ETD
cross−linker cleavage
intensity
mass
0e+00
2e+05
4e+05
6e+05
8e+05
1e+06
500
1000
1500
B+XL_L
A+XL_S
B+XL_S
A+XL_L
y4
b3
c3
z1
z2
z3
z4
y4
b2
c3
c4
c5
c6
z4
z5
z5
z6
DAGKQGK+XL_S
A_b_ion
A_y_ion
prec
b3+
y1+
m/z
DAGKQGK+XL_L
HKQFWR+XL_S
B_b_ion
B_y_ion
y4+
b2+
y5+
y2+
prec
y3+
b4+
b5+
y1+
HKQFWR+XL_L
b2+
y4+
y5+
y5++
b5+
y1+
b2++
b3++
y2+
y3+
b4+
